# LiftoffTools: a toolkit for comparing gene annotations mapped between genome assemblies

**DOI:** 10.12688/f1000research.124059.1

**Published:** 2022-10-28

**Authors:** Alaina Shumate, Steven Salzberg

**Affiliations:** 1 Biomedical Engineering, Johns Hopkins University, Baltimore, MD, 21218, USA; 2 Center for Computational Biology, Whiting School of Engineering, Johns Hopkins University, Baltimore, MD, 21211, USA; 3 Computer Science, Johns Hopkins University, Baltimore, MD, 21218, USA; 4 Biostatistics, Bloomberg School of Public Health, Johns Hopkins University, Baltimore, MD, 21205, USA

**Keywords:** Bioinformatics, Genome annotation, Genomics

## Abstract

In 2020 we published Liftoff, which was the first standalone tool specifically designed for transferring gene annotations between genome assemblies of the same or closely related species. While the gene content is expected to be very similar in closely related genomes, the differences may be biologically consequential, and a computational method to extract all gene-related differences should prove useful in the analysis of such genomes. Here we present LiftoffTools, a toolkit to automate the detection and analysis of gene sequence variants, synteny, and gene copy number changes.  We provide a description of the toolkit and an example of its use comparing genes mapped between two human genome assemblies.

## Introduction

Liftoff ( Shumate and Salzberg, 2021) is a computational tool specifically designed for mapping gene annotations from a reference assembly to a target assembly of the same or closely related species. Liftoff uses sequence alignment software to align the complete exon-intron structure of each annotated transcript from a source to a target, and it can also map virtually any other feature specified as an interval along the genome. It also includes a method to find additional copies of genes that might be present in higher copy numbers in the target genome. After lifting genes over, one of the first questions that many researchers have is how the sets of genes compare between the reference assembly and the target, and in particular whether any of the differences are biologically consequential.

Here we introduce LiftoffTools, a toolkit to compare genes mapped from one assembly to another. LiftoffTools includes three different modules. The first identifies changes in protein-coding genes and their effects on the corresponding genes, including simple amino acid changes as well as more-serious alterations. The second compares the gene synteny ( *e.g.*, the preservation of gene order along the chromosomes), and the third clusters genes into groups of paralogs to evaluate gene copy number gain and loss. While LiftoffTools is designed to analyze the output of Liftoff, it is also compatible with the output of other annotation transfer tools such as UCSC liftOver ( Kuhn *et al.,* 2013). Here we provide a description of each module as well as results comparing genes in the GRCh38 human reference genome mapped onto CHM13, the first truly complete human genome ( Nurk *et al.,* 2022).

## Methods

The inputs required for all three modules of LiftoffTools are the sequences of the reference and target assemblies (in FASTA format), and the annotations of the reference and target assemblies (in GFF3 or GTF format). The target annotation can be derived from other lift-over tools besides Liftoff, as long as the feature IDs in the reference and target annotations are the same. All three modules can be run with the following command:

liftofftools all -r reference.fasta -t target.fasta -rg reference.gff3 -tg target.gff3

Each module can also be run separately as detailed on GitHub.

### Operation

LiftoffTools is designed and implemented in Python 3 (requires 3.6 or higher) and is easily installable with PyPi (pip install liftofftools) and bioconda (conda install -c bioconda liftofftools). Details on how to run LiftoffTools are available on the GitHub page.

### Variants

The variants module calculates the sequence identity between transcripts in the reference genome and the corresponding transcripts in the target genome. For protein-coding genes, the module identifies variants and their effect on the translated amino acid sequences. The first step in the module will globally align the nucleotide sequences of the reference transcripts to the target transcripts using the Needleman-Wunsch algorithm implemented by Parasail ( Daily, 2016), which is a single instruction/multiple data (SIMD) C library for sequence alignment. If the transcript has an annotated protein-coding sequence (a CDS feature), we align the protein sequences again using Parasail. We then identify mismatches and gaps in the alignments and evaluate the effects on the protein sequence. The potential effects we look for are synonymous mutations, nonsynonymous mutations, in-frame deletions, in-frame insertions, start codon loss, 5′ truncations, 3′ truncations, frameshifts, and stop codon gain. For all transcripts we output the percent identity at the nucleotide level, and for protein-coding transcripts we also output the protein percent identity and the variant effect if applicable. If there is more than one variant, we report only the most severe. For example, if a transcript has a synonymous mutation and a frameshift mutation, we output ‘frameshift’ for that transcript as this would be more disruptive to gene function.

### Synteny

The synteny module compares the gene order in the reference annotation to the order in the target annotation. The genes present in both annotations are sorted first by chromosome and then by start coordinate in each annotation. Each gene is then plotted as a point on a 2D plot where the x-coordinate is the ordinal position ( *e.g.*, 1 ^st^, 2 ^nd^, 3 ^rd^, etc.) in the reference genome and the y-coordinate is the ordinal position in the target genome. The color of the point corresponds to the sequence identity between the corresponding genes, where green indicates higher identity and red indicates lower identity. Note this color feature is only available for target annotations created by Liftoff which have the sequence identity information in the GTF/GFF3. The plot and a file with the ordinal positions and sequence identities of each gene is output. The user also has the option to calculate the edit distance between the reference order and the target order.

### Clusters

This module clusters the genes into paralogous groups to evaluate gene copy number gain and loss. LiftoffTools first invokes MMSeqs2 ( Steinegger and Söding, 2017) to cluster the reference gene sequences. MMSeqs2 clusters the amino acid sequences of the protein-coding genes, and the nucleotide sequences of noncoding genes. For each gene we select only the longest isoform to be included in the clustering. For genes to be considered copies and be clustered together, they must be at least 90% identical across 90% of both of their lengths, although these parameters can be adjusted by the user. After clustering the reference genes, we create the target gene clusters by first iterating through each reference cluster and removing any gene that failed to map to the target genome. Next, if Liftoff was run with the -copies option to identify extra gene copies in the target genome, we add the extra copies to the same cluster as their closest paralog. For each cluster, we output the number of reference genes and the number of target genes belonging to that cluster as well as the gene IDs of the cluster members.

## Results

To illustrate the use of these tools, we used them to compare the human annotation on the current reference genome, GRCh38, to the same annotation when mapped onto the first-ever complete human genome, CHM13 ( Nurk *et al.,* 2022). We first mapped the human annotation onto CHM13 by running Liftoff v1.6.3 (with options -copies -sc 0.95 -polish) to map genes from RefSeq release 110 ( O’Leary *et al.,* 2016) from GRCh38 onto CHM13v2.0. (This annotation is available on the Johns Hopkins Center for Computational Biology website) We then ran each module of LiftoffTools on the resulting CHM13 annotation.

### Variants

Running the variants module on GRCh38 and CHM13, we found that out of 130,316 protein-coding transcripts in GRCh38, 77,109 CHM13 transcripts were identical, 421 failed to map, and 52,669 had variants with the effects shown in Table 1. The vast majority of these effects were either simple amino acid changes or insertion/deletions (indels) that preserved the reading frame; only 932 of the variants had a major effect on the translated protein sequence.

**Table 1.** Effects of sequence differences on protein-coding transcripts and the number of transcripts affected in CHM13 identified by the LiftoffTools variants module. In-frame changes refer to insertions or deletions that are a multiple of 3 in length. Truncations are variants that shorten the protein sequence by removing either the 5′ or 3′ end of the transcript including the start or stop codon. Start codon loss variants are point mutations in the start codon, and stop codon gain variants are point mutations that result in a premature stop codon.

Variant effect	Number of transcripts
None (synonymous)	21,823
Non-synonymous	28,507
In-frame deletion	744
In-frame insertion	663
Start codon lost	117
5’ truncation	1
3’ truncation	7
Frameshift	718
Stop codon gained	206

### Synteny

**Figure 1.**
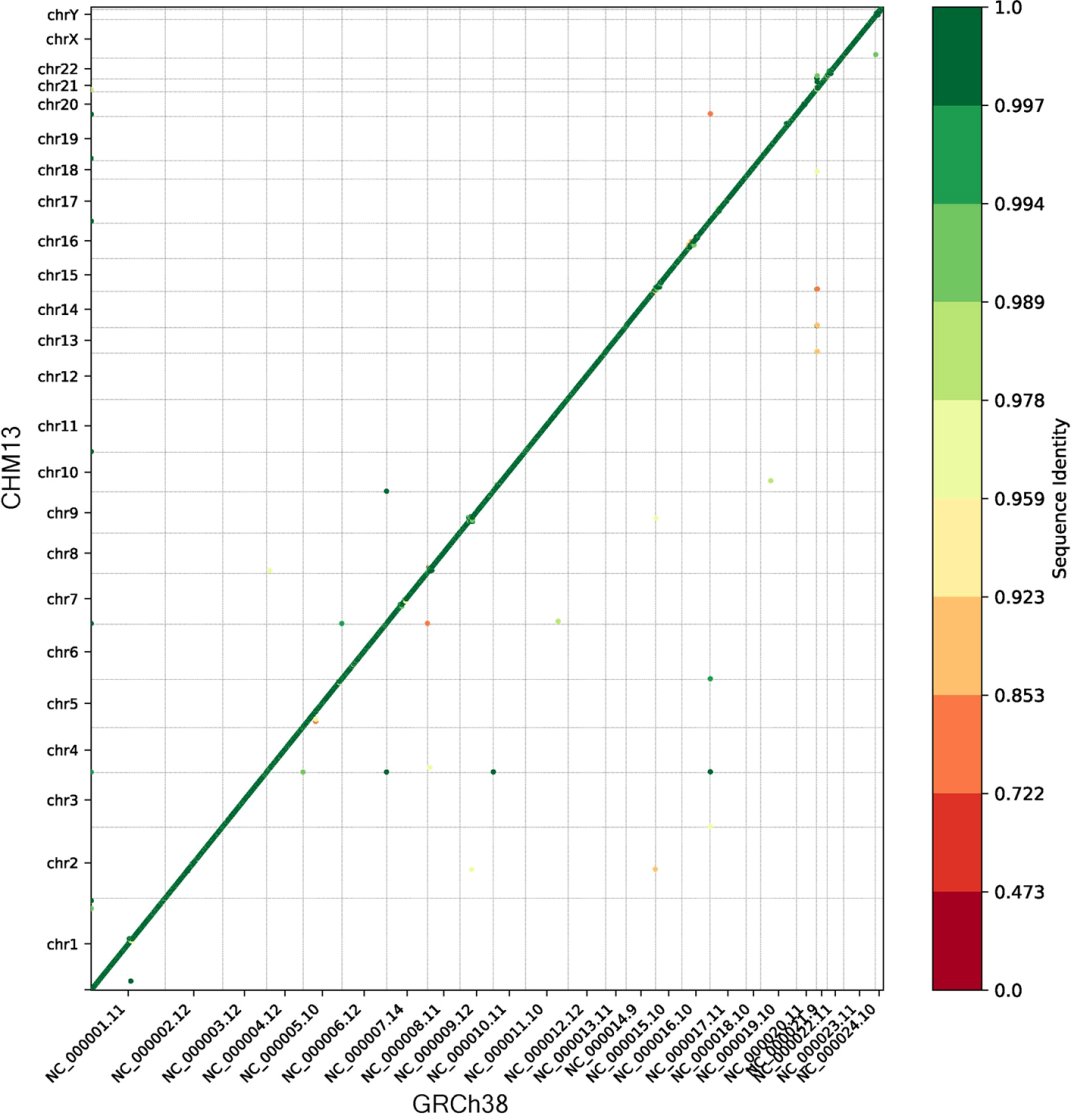
Dot plot showing the ordinal position of each gene in GRCh38 on the x-axis and the ordinal position in CHM13 on the y-axis. The color of each point indicates the sequence identity, and the gray lines separate the chromosomes.

We ran the synteny module to compare the gene order of CHM13 to GRCh38. The dot plot in Figure 1 shows that the vast majority of genes were collinear and nearly identical in sequence, as expected. The small number of genes which were not collinear generally mapped with a lower sequence identity, suggesting they may have been mapped to a different (non-syntenic) copy of a gene in a multi-gene family.

### Clusters

The clusters module found 5,213 genes in GRCh38 with at least one paralog that met the 90% sequence identity and alignment length minimums. These 5,213 genes were grouped into 1,629 clusters with copy numbers ranging from two to 66. In CHM13, 8,356 genes had at least one paralog. These copies were grouped into 2,089 clusters with copy numbers ranging from two to 228. (Note that the ribosomal DNA gene is the largest cluster, and most copies of this gene are not present in the GRCh38 assembly.) Among clusters with a copy number of at least 2 in GRCh38, 134 clusters had fewer gene copies in CHM13 resulting in a total loss of 188 gene copies. A total of 715 clusters had more copies in CHM13 resulting in a total gain of 3,035 gene copies.

## Conclusions

Liftoff gave us the ability to easily map genes between closely related genomes, but further analysis is required to identify similarities and differences between the genes in each assembly that may be biologically important. LiftoffTools enables this analysis by automating the comparison of protein-coding variants, gene synteny, and gene copy loss and gain. Here we provided an example demonstrating the use of LiftoffTools to compare genes mapped between two human assemblies, and we hope this set of tools will be useful for a wide diversity of assembled genomes from species across all domains of life.

## Software availability

Source code available from: https://github.com/agshumate/LiftoffTools

Archived source code as at time of publication: https://doi.org/10.5281/zenodo.6967163 ( Shumate, 2022)

License: GNU GPL v3

## Data availability

### Underlying data

GRCh38 sequence: https://ftp.ncbi.nlm.nih.gov/genomes/all/GCF/000/001/405/GCF_000001405.26_GRCh38/GCF_000001405.26_GRCh38_genomic.fna.gz

CHM13 sequence: https://ftp.ncbi.nlm.nih.gov/genomes/all/GCF/009/914/755/GCF_009914755.1_T2T-CHM13v2.0/GCF_009914755.1_T2T-CHM13v2.0_genomic.fna.gz

CHM13 annotation: https://ccb.jhu.edu/T2T.shtml or ftp://ftp.ccb.jhu.edu/pub/data/T2T-CHM13/chm13v2.0_RefSeq_Liftoff_v3.gff3

(Note: The CHM13 annotation has been updated to v4 since the submission of this manuscript)
