# Supplementary material for: LiftoffTools: a toolkit for comparing gene annotations mapped between genome assemblies
Source: F1000Res. 2024 Apr 29;11:1230. Originally published 2022 Oct 28. [Version 2] doi: 10.12688/f1000research.124059.2 (PMC11137477; doi:10.12688/f1000research.124059.2)
Supplement: Peer review history

# Reviewer report: Reviewer response for version 2

**DOI:** 10.5256/f1000research.164429.r270095

**Published:** 2024-05-29

**Authors:** Adam Frankish

**Affiliations:** 1 European Bioinformatics Institute, Wellcome Genome Campus, European Molecular Biology Laboratory, Cambridge, UK

Thanks to the authors for their clear responses to questions and updates to the manuscript.

Are the conclusions about the tool and its performance adequately supported by the findings presented in the article?

Yes

Is the rationale for developing the new software tool clearly explained?

Yes

Is the description of the software tool technically sound?

Yes

Are sufficient details of the code, methods and analysis (if applicable) provided to allow replication of the software development and its use by others?

Yes

Is sufficient information provided to allow interpretation of the expected output datasets and any results generated using the tool?

Partly

Reviewer Expertise:

Gene annotation

I confirm that I have read this submission and believe that I have an appropriate level of expertise to confirm that it is of an acceptable scientific standard.

# Reviewer report: Reviewer response for version 1

**DOI:** 10.5256/f1000research.136230.r212791

**Published:** 2023-11-29

**Authors:** Mark Borodovsky

**Affiliations:** 1 School of Computational Science and Engineering, Wallace H. Coulter Department of Biomedical Engineering, Georgia Institute of Technology, Atlanta, Georgia, USA

The manuscript is well written and presents a useful computational tool for comparison of gene annotations between genome assemblies.

 I have two minor comments for the Methods section.

 Variants

 Do transcripts include UTRs?

 If yes, the applicability is limited to genomes with annotated UTRs, if no – the term transcript should be defined as such.

 Clusters

 In the sentence: 

 “Next, if Liftoff was run with the -copies option to identify extra gene copies in the target genome, we add the extra copies to the same cluster as their closest paralog.” 

 The meaning of the “-copies” option, the difference with the default run, was not described.

Are the conclusions about the tool and its performance adequately supported by the findings presented in the article?

Yes

Is the rationale for developing the new software tool clearly explained?

Yes

Is the description of the software tool technically sound?

Yes

Are sufficient details of the code, methods and analysis (if applicable) provided to allow replication of the software development and its use by others?

Yes

Is sufficient information provided to allow interpretation of the expected output datasets and any results generated using the tool?

Yes

Reviewer Expertise:

Bioinformatics, Genome Analysis

I confirm that I have read this submission and believe that I have an appropriate level of expertise to confirm that it is of an acceptable scientific standard.

# Response

**Published:** 2024-03-29

**Authors:** Alaina Shumate

**Affiliations:** Johns Hopkins University, USA

**Comment 1**

 Variants

 Do transcripts include UTRs? If yes, the applicability is limited to genomes with annotated UTRs, if no – the term transcript should be defined as such.

**Response 1**

 The mRNA transcripts are aligned which we derive by concatenating all of the ‘exon’ features in the GFF3 annotation.  Some annotations include UTRs in the 5’ or 3’ exons. In these cases, yes, the UTRs are included. Others annotate them separately as their own feature upstream or downstream of exons. In these cases, they are not included. While the full transcript is used to calculate sequence identity, variants are only called within the amino acid sequence, so the module can be applied to genomes with or without annotated UTRs with no effect on the number or types of variants reported.

**Comment 2**

 Clusters

 In the sentence: 

 “Next, if Liftoff was run with the -copies option to identify extra gene copies in the target genome, we add the extra copies to the same cluster as their closest paralog.” 

 The meaning of the “-copies” option, the difference with the default run, was not described.

**Response 2**

 I have updated the manuscript to include the text below for clarification:

*“Next, if Liftoff was run with the -copies option to identify extra gene copies in the target genome, we add the extra copies to the same cluster as their closest paralog. If Liftoff was run without the -copies option, no extra gene copies will be present in the target annotation, and thus the clusters module will only report instances of copy number loss.”*

# Reviewer report: Reviewer response for version 1

**DOI:** 10.5256/f1000research.136230.r154534

**Published:** 2022-12-01

**Authors:** Adam Frankish

**Affiliations:** 1 European Bioinformatics Institute, Wellcome Genome Campus, European Molecular Biology Laboratory, Cambridge, UK

This manuscript describes a set of tools to analyze gene annotation that has been mapped between one genomic sequence and another closely related genomic sequence. The methods described build on the widely adopted Liftoff annotation transfer tool developed by the same group.

 The development of LiftoffTools is clearly relevant and timely as we enter an era of rapidly increasing numbers of high quality genome sequences suitable for comparison to species reference genome assemblies that are generally the focus of gene annotation effort. The description of the tools is sound and we were able to run the code with the instructions provided and generate the correct/expected output. The code is written entirely in Python and looks clear and well-organized.

 There is broadly sufficient information to allow interpretation of the results, although a little more guidance might be useful, for example, adding annotated examples of real data from Variant and Cluster analysis to guide users.

**Comments:**

- "... *as long as the feature IDs in the reference and target annotations are the same. All three modules can be run with the following command*" - Is this somewhat inflexible? Not all transfer methods will preserve feature ID identically across different assemblies as they seek to avoid storing two or more features with identical IDs but different properties (sequence/length/etc). Is it possible to accommodate methods that take this approach?
- It is not clear from the manuscript or the supporting information in https://github.com/agshumate/LiftoffTools/blob/master/README.md how LiftoffTools handles genes that are LoF on genome 1 but functional on genome 2. Can these genes and their 'rescuing' variation be identified?
- While the list of variant consequences is comprehensive for the annotated CDS, it would be useful to add other LoF consequences such as disruption of core splice site to the analysis.
- It would also be useful to specifically state the ranking of consequences in the /README.md file for genes with multiple transcript-affecting variants as only the most significant is provided in the variation output file.
- Similarly, as only one variant is reported, does LiftoffTools identify (and/or flag) corrective variation e.g a second frameshift that compensates for an earlier frameshift and restores the CDS with a small aa change?
- In the calculation of cluster gain/loss, are haplotypic duplicated pseudogenes considered? i.e. is loss only deletion/absence of the gene or is loss (or gain) of function included as well? An example with real data in /README.md could be helpful.
- What is reported for variants in genes that are missing or partial on GRCh38 where it is used as a reference? An example with real data in /README.md could be helpful.
- The CHM13 GFF and FNA (fasta) files appear to have different chromosome names, which threw an error when running the code.

Are the conclusions about the tool and its performance adequately supported by the findings presented in the article?

Yes

Is the rationale for developing the new software tool clearly explained?

Yes

Is the description of the software tool technically sound?

Yes

Are sufficient details of the code, methods and analysis (if applicable) provided to allow replication of the software development and its use by others?

Yes

Is sufficient information provided to allow interpretation of the expected output datasets and any results generated using the tool?

Partly

Reviewer Expertise:

Gene annotation

I confirm that I have read this submission and believe that I have an appropriate level of expertise to confirm that it is of an acceptable scientific standard, however I have significant reservations, as outlined above.

# Response

**Published:** 2024-03-29

**Authors:** Alaina Shumate

**Affiliations:** Johns Hopkins University, USA

**Comment 1**

 Is this somewhat inflexible? Not all transfer methods will preserve feature ID identically across different assemblies as they seek to avoid storing two or more features with identical IDs but different properties (sequence/length/etc). Is it possible to accommodate methods that take this approach?

**Response 1**

 While it would be great to accommodate all lift-over tools, without the preservation of feature IDs, identifying the reference/target gene equivalents for comparison becomes a non-trivial task. LiftoffTools was specifically designed as a post-processing toolkit for the output of Liftoff , and in our experience, other commonly used tools such as UCSC liftOver do indeed preserve feature IDs. Furthermore, the GFF3 specification states that all feature IDs must be unique.  If a transfer method is changing IDs to eliminate duplicates, this is an issue of the reference annotation failing to adhere to the GFF3 specifications and should be addressed prior to mapping annotations.

**Comment 2**

 It is not clear from the manuscript or the supporting information in https://github.com/agshumate/LiftoffTools/blob/master/README.md how LiftoffTools handles genes that are LoF on genome 1 but functional on genome 2. Can these genes and their 'rescuing' variation be identified?

**Response 2**

 Currently, LiftoffTools does not identify ‘rescuing’ variants. In our particular case, we used the RefSeq annotation as a reference, which requires a valid open reading frame to annotate a coding sequence; therefore, there are no LoF coding sequences annotated in the reference to be mapped. We recognize that this is not true for all reference annotations; many include annotations of coding sequences without a valid open reading frame. There are varying schools of thought on whether this is acceptable, but regardless, we do agree that identifying rescuing or gain of function variants would be useful, and we will consider implementing that feature in the future. We have updated the manuscript to say “the module identifies variants that have a neutral or deleterious effect on the translated amino acid sequences in the target genome.” The README has also been updated.**

**Comment 3**

 While the list of variant consequences is comprehensive for the annotated CDS, it would be useful to add other LoF consequences such as disruption of core splice site to the analysis.

**Response 3**

 We do agree that splice site variants would be useful to include, but we are currently only aligning and looking at variants in the mRNA due to computational limitations. Performing Smith-Waterman alignment on transcript sequences including splice-sites and introns would require significant computational resources for human and other eukaryotic genomes. Even with a very fast implementation of the Smith-Waterman alignment, aligning just the mRNA and amino acid sequences is the computational bottleneck of the LiftoffTools pipeline. While faster alignment methods could alleviate this, they would not have the same accuracy as Smith-Waterman, which we feel is necessary for accurately identifying the position and type of variants. We have edited the manuscript to specifically state that we are aligning mRNA sequences.

**Comment 4**

  It would also be useful to specifically state the ranking of consequences in the /README.md file for genes with multiple transcript-affecting variants as only the most significant is provided in the variation output file.

**Response 4**

 Thank you for the suggestion. I have updated the README.md accordingly.

**Comment 5**

 Similarly, as only one variant is reported, does LiftoffTools identify (and/or flag) corrective variation e.g a second frameshift that compensates for an earlier frameshift and restores the CDS with a small aa change?

**Response 5**

 We do not report this explicitly. The intent of the variants module is to provide high level summary information about how many genes were disrupted by variants rather than identifying every variant in every gene. We do however include the amino-acid level sequence identity information, so compensatory frameshifts can be inferred if a sequence is reported to have a frameshift but also retains a high sequence identity to the reference protein. We have added the following text to the manuscript to capture these points.

*“While there may be multiple variants within a transcript, the intent of this module is to summarize the functional consequences of variation; therefore, if there is more than one variant, we report only the most severe. For example, if a transcript has a synonymous mutation and a frameshift mutation, we output ‘frameshift’ for that transcript as this would be more disruptive to gene function. Combining the sequence identity information with the variant effect can provide further insights into the severity of the variant. For example, a gene with a frameshift near the 3’ end or a gene with a compensatory frameshift nearby will have a high percent identity at the amino acid level and may still retain function.”*

**Comment 6**

 In the calculation of cluster gain/loss, are haplotypic duplicated pseudogenes considered? i.e. is loss only deletion/absence of the gene or is loss (or gain) of function included as well? An example with real data in /README.md could be helpful.

**Response 6**

 This is a good point. Liftoff intentionally avoids annotating extra gene copies in the target genome that are processed pseudogenes by including introns in the initial alignment step. Therefore, when working with the output of Liftoff, it is not something we need to consider. If a different annotation tool was used that does annotate pseudogenes, they will get clustered with their paralogs even if they are not functional. As previously mentioned, LiftoffTools was designed to be used in conjunction with Liftoff, so we have not considered a strategy for removing non-functional pseudogene copies from the clusters.

**Comment 7**

 What is reported for variants in genes that are missing or partial on GRCh38 where it is used as a reference? An example with real data in /README.md could be helpful.

**Response 7**

 Variants are only reported for genes that are in both GRCh38 and CHM13. We state in the manuscript

 “ *The variants module calculates the sequence identity between mRNA transcripts in the reference genome and the corresponding transcripts in the target genome…”*

 A limitation of lifting over gene annotations is that a partial reference gene will likely also be annotated as partial in the target annotation. There are various lift-over algorithms/strategies; however, they generally rely on converting the start and end coordinates of the gene from reference to target.  If the start-end range is only a partial gene, only that part of the gene will be lifted over. In these cases, they will be reported as either a 5’ truncation or a 3’ truncation based on the presence or absence of start and stop codons.

**Comment 8**

 The CHM13 GFF and FNA (fasta) files appear to have different chromosome names, which threw an error when running the code.

**Response 8**

 Thank you for bringing this to our attention. The link to the fasta file has been replaced to a file with the same chromosome names.
